# Bromide of Potassium in Hypertrophy of the Spleen

**Published:** 1875-03

**Authors:** 


Bromide of Potassium in Hypertrophy of the Spleen.—*New Remedies*, Jan., 1875, p. 23, quotes from *The Clinic* of Sept. 15, 1874, an article by Dr. Ch. Bernard in *Bul. Gen. Therap.* of which the following is an abstract:

Enlargements of the spleen are very common in malarial districts in Algeria, often compressing the intestines, embarrassing the circulation and determining serious accidents, sometimes death. The disease is also very obstinate, resisting every form of medication known to the present day. The following treatment has not failed once in the thirty-eight times it was used:

The volume of the spleen augments not only after the access of fever. There are hypertrophies which have never been preceded by the least febrile movement. In these quinia produces no effect. There are others caused by and subsiding with intermittents, which, however, do not arrive at a perfect cure, and each recurrence increases the splenic engorgement. In these we cannot rely on febrifuges but must have recourse to a medication *degogeante, resolutive.* There are also cases where quinia entirely cures the fever but in which the engorgement remains untouched.

Splenic intumescence sometimes is enormous, hard, painful to pressure, even to touch, gives a sense of resistance, reaches beyond the linea alba, crowds back the intestines, pushes up the heart and left lung, sometimes interfering with their functions, takes up the whole left hypochondrium and occasionally a great part of the pelvis. The following case furnished M. Bernard the indications that led to his new treatment, after he had frequently failed in obtaining satisfactory results:

M. A——, a very intelligent man, had inhabited the plain of the Mitidja for fifteen or twenty years, during which he had often been attacked with intermittent fever, which had exhausted his constitution. He had several relapses with cerebral accidents, very painful and very disturbing, with delirium, nervous agitations, etc. The spleen was enormous, hard and greatly hypertrophied. Different kinds of treatment had often been ineffectually tried.

In the last year he was seized with an accession of very violent nervous attacks, for which three grammes (forty-five grains) in one potion was given daily. On the tenth day the spleen was diminished one-half. The treatment was continued twenty-five days up to complete restoration of spleen. In repeating the experiment the same result was always obtained.

We may secure complete resolution of intumescence of the spleen in different conditions by the bromide of potassium in doses of one gramme daily, in an infusion of orange leaves, for fifteen or twenty days. It is rarely necessary to prolong the treatment beyond thirty days.

His last case was this:

An Arab of Turkish origin had an enormous belly resistant, very painful in the left hypochondrium, and most pronounced anæmia; all these conditions existing for six years. After a month of treatment by the bromide of potassium he resumed his occupation. Six months have now passed, and the left hypochondrium has presented its normal aspect and state. There has been no recurrence.

M. Bernard says hypertrophies of the liver yield as readily, or at least are much improved, by this medication in the same doses.

